# Electromagnetic source reconstruction for group studies

**DOI:** 10.1016/j.neuroimage.2008.06.022

**Published:** 2008-10-01

**Authors:** Vladimir Litvak, Karl Friston

**Affiliations:** The Wellcome Trust Centre for Neuroimaging, University College London, UK

**Keywords:** Hierarchical Bayes, Source reconstruction, EEG, MEG, Restricted maximum likelihood, Automatic relevance determination

## Abstract

The aim of this paper is to describe a simple procedure for electromagnetic (EEG or MEG) source reconstruction, in the context of group studies. This entails a simple extension of existing source reconstiruction techniques based upon the inversion of hierarchical models. The extension ensures that evoked or induced responses are reconstructed in the same subset of sources, over subjects. Effectively, the procedure aligns the deployment of reconstructed activity over subjects and increases, substantially, the detection of differences between evoked or induced responses at the group or between-subject level.

## Introduction

There has been considerable progress over the past few years in reconstructing electromagnetic source activity from channel data (*e.g*., Baillet and Garnero, 1997; Phillips et al., 2002; Phillips et al. , 2005; Sato et al., 2004; Serinagaoglu et al., 2005; Trujillo-Barreto et al., 2004; Jun et al., 2006; Mattout et al., 2006; Nummenmaa et al., 2007; Wipf and Nagarajan, in press). These advances rest upon reformulating classical inversion approaches within a Bayesian framework and then using hierarchical or empirical Bayes (Kass and Steffey, 1989; Phillips et al., 2005; Wipf and Nagarajan, in press) to optimise both the estimated sources *and the constraints or priors themselves*. This optimisation uses standard sampling or variational schemes to provide the most likely distribution of source activity, given observed data. While these reconstructions are optimal for data from a single subject or session, they are not optimal for detecting group differences. This is because the hierarchical model does not include a between-subject level and is therefore unable to exploit the fact that the same paradigm was repeated over subjects or sessions. The analysis described in this paper is based on two established approaches: the summary-statistic approach to hierarchical models and the use of a canonical mesh in source reconstruction.

### The summary-statistic approach

The problem of making inferences about population responses is resolved by hierarchical models. In classical statistics, these are known as random or mixed-effects models that include random effects at the within and between-subject level. In Bayesian inference these are known as parametric empirical Bayes models, where between-subject variations in parameter estimates furnish constraints on within-subject estimation. Usually, these random effects or hierarchical models are inverted using data from all the subjects analysed. An alternative approach is to optimise the parameters of subject-specific models separately and use these optimised parameters as summary-statistics, which are then analysed in a second-level or between-subject model. This can be repeated until the top level of the hierarchical model is reached. Provided the models for each subject are the same, the summary-statistic approach gives, in expectation, exactly the same results as a full mixed-effects analysis. Furthermore, even if there are differences between the models at the single-subject level; the differences between the summary-statistic approach and a mixed-effects analysis are trivial (see Friston et al., 2005 for example in fMRI). In neuroimaging people generally use the summary-statistic approach because, computationally and conceptually, it is much simpler to implement; and because it avoids handling all the data from all the subjects at the same time. This is important in neuroimaging, where the size of datasets can be very large.

In the analysis of group EEG and MEG studies, we anticipate that people will want to use a summary-statistic approach, in which a summary of evoked responses (within some time and frequency window) for each subject is optimised at the within-subject level and then passed to a simple ANOVA model at the second level, to produce statistical parametric maps (SPM) of regionally significant differences among trials. In this particular application, one would use Bayesian estimators as the summary-statistic at the within-subject level and classical statistics for inference at the between-subject level. However, for this two-stage procedure to work, the reconstructed activity at a particular source in one subject should correspond to the same source in the other subjects. This brings us to the notion of a canonical mesh.

### The canonical mesh

To facilitate group-level analyses, we introduced the idea of a canonical mesh (Mattout et al., 2007), which ensures that activity is reconstructed in the same source space over subjects (Talairach and Tournoux, 1988). Briefly, the forward model for each subject starts with a canonical mesh, defining a lattice of sources on the cortical surface. This mesh is then warped using an inverse spatial normalization so that the canonical mesh is roughly in the same place as the subject's cortical sheet. This means that, after inversion of the ensuing forward model, reconstructed activity can be assigned to the same sources over subjects. One can then project this two-dimensional manifold into a three-dimensional image and proceed in the usual way to make inferences about regionally-specific effects using SPM. However, for this to be efficient, one needs to suppress inter-subject variability in the spatial deployment of reconstructed activity. This can arise from the underdetermined aspect of the inverse problem and calls for formal constraints on single-subject reconstructions. These constraints or hyperpriors are the subject of this technical note.

### Hyperpriors

The importance of hierarchical or empirical models for single-subject data rests upon optimising not just the parameters of the model (like source activity) but also the priors themselves. This optimisation proceeds under hyperpriors, which can be regarded as priors or constraints on the prior expectations about the pattern of source expression. Critically, the hyperpriors can also be optimised to select the best hyperprior or models (*c.f*., model selection). A simple example of different models would be the distinction between a conventional minimum norm prior and multiple sparse priors. In the former, the prior constraint is that activity has exactly the same variance at every source and, furthermore, is independent. This is encoded by a prior covariance on source space that corresponds to the identity matrix; *i.e*., *Q*_1_^*U*^ = I. Conversely, in multiple sparse priors the hyperprior is that activity can be expressed in multiple patches or covariance components, *Q*_1_^*U*^,…,*Q_m_^U^*, each of which has an associated hyperparameter (Friston et al., 2008a). The difference between minimum norm and multiple sparse prior models rests on the form or number of prior covariance components or, equivalently, the number of hyperparameters. These hyperparameters can be optimised using a variety of techniques. Recently we introduced a modelling approach that is formally identical to restricted maximum likelihood for optimising hundreds of hyperparameters, under multiple sparse priors. The sparse aspect speaks to the fact that many of these prior covariance components are unnecessary or irrelevant and can be switched off (*c.f*., automatic relevance determination; ARD; Neal, 1998). This automatic hyperprior or model selection depends on choosing the right objective function (a variational bound on the model's log-evidence that includes weakly informative hyperpriors). An alternative approach to optimising the hyperpriors is to use a greedy search as described in Friston et al. (2008b). The distinction between ARD and greedy search optimisation of models is relatively simple: in ARD one assumes that every hyperparameter has a different value. These hyperparameters are then optimised so that many fall to zero, leaving a small number of relevant or important empirical priors (Tipping, 2001). In a greedy search, one starts by assuming all the hyperparameters have the same value and then splits the set of hyperparameters recursively, until the objective function stops increasing (Friston et al., 2008b). We will see examples of minimum norm, ARD and greedy search schemes later.

The particular hyperprior we propose here imposes consistency on the source reconstruction over subjects or sessions. It is a formal constraint that assumes that the prior variance at any particular source in any particular subject can be factorised into a subject-specific and source-specific term. In other words, we assume that the unknown empirical prior variance is proportionally the same over sources but the variance *per se* is scaled in a subject-specific fashion. This simple assumption allows us to estimate source-specific hyperparameters by pooling the data from all trial types and subjects to optimise the form of the prior covariance over sources. This empirical spatial prior then enters a series of single-subject inversions, which optimises the weight afforded to this prior, in relation to subject-specific measurement noise. This formal constraint ensures that the source reconstruction in each individual analysis is confined to the same sources, without biasing the parameter estimates themselves. This means that we can use the parameter estimates as summary-statistics for valid inference at the between-subject level. Note that this does not represent an inversion of a full hierarchical model. Our objective is to finesse the first-level Bayesian inversion to provide useful summary-statistics for classical inference at the second level. By pooling the data to estimate empirical priors on source space, we can meet this objective simply and effectively.

This paper comprises two sections. In the first, we present the details of the model inversion. This inversion has two stages. The first optimises the empirical prior covariance over subjects. The second entails inverting the data from each subject using the empirical prior from the pooled analysis. We will briefly describe the implicit hierarchical models and the optimisation scheme that we use. In the second section, we will present comparative analyses of between-subject or group-level inference with and without between-subject constraints. We would also take the opportunity to compare the performance of three hyperpriors: a minimum norm hyperprior with a single hyperparameter (this can be regarded as the baseline hyperprior). Second, we will look at multiple sparse priors that have been optimised using ARD. Finally, we will consider the hyperpriors furnished by a greedy search over successive partitions of multiple sparse priors. The formal details concerning these sparse priors will be found in Friston et al. (2008a) and the technical details of the greedy search will be found in Friston et al. (2008b). In short, we will compare three hyperprior models of source activity with and without between-subject constraints. The outcome measures we use reflect the quality of each model at the within-subject level and the efficiency of detecting group effects at the between-subject level. The within-subject measure is simply the log-evidence for each of the six (2 × 3) models for each subject (actually five models because the minimum norm is the same with and without constraints — see below). For the between-subject analysis we use two measures based on between-subject SPMs from each of the models. These measures reflect sensitivity (peak *t*-values) and spatial accuracy (location of peaks). The dataset we use involves a simple sensory evoked response following median nerve stimulation. We chose these data because the location of the underlying sources is well known and allows us to interpret our comparative analyses with greater confidence.

## Theory

### Covariance function models

We start with a hierarchical generative model for data $Y_{i}=R^{c\times n}$ over *c* channels and *n* peristimulus time-bins, from *i *= 1,…,*N* trials or subjects^1^. We assume that random effects on the sources factorise onto subject-specific *P*(*ν*^*P*^) and source-specific terms *U*(*ν*^*U*^), such that their covariance is *P* ⊗ *U*. Here, $\nu^{•}$ are unknown scale parameters that control the covariance or amplitude of random effects in source space. This assumption engenders a probabilistic generative or forward model with empirical (*i.e*., hierarchical) priors on sources *J*. These sources are mapped to channels by subject-specific lead-field or gain matrices, *L*_1_,…,_*N*_ to give the following linear model; *Y *= *LJ *+ *ɛ*

(1) $$\left[\begin{array}{c} Y_{1}\\ \vdots \\ Y_{N} \end{array}\right]=\left[\begin{array}{ccc} L_{1} & & \\ & \ddots & \\ & & L_{N} \end{array}\right]\left[\begin{array}{c} J_{1}\\ \vdots \\ J_{N} \end{array}\right]+\left[\begin{array}{c} {ɛ}_{1}\\ \vdots \\ {ɛ}_{N} \end{array}\right]$$

Where, under Gaussian assumptions about random effects,

(2) $$\begin{array}{c} p\left(Y|J,\lambda \right)=N\left(LJ,V,C\right)\\ p\left(J\right)=N\left(0,V,P⊗U\right)\\ p\left(\lambda \right)=N\left(\eta ,R\right)\\ C=\mathrm{diag}\left(\nu_{1}^{C},\ldots ,\nu_{N}^{C}\right)⊗Q^{C}\\ U=\nu_{1}^{U}Q_{1}^{U}+\ldots +\nu_{M}^{U}Q_{M}^{U}\\ P=\mathrm{diag}\left(\nu_{1}^{P},\ldots ,\nu_{N}^{P}\right)\\ \nu =\text{exp}\left(\lambda \right) \end{array}$$

Here, *p*(*ɛ*) = *N*(0,*V*,*C*) denotes a zero-mean Gaussian density on a matrix with temporal correlations, *V* and spatial covariances among channels, *C*. In this model, *Q*^*C*^ represents a single covariance component over channels, which is scaled by a subject-specific covariance parameter or hyperparameter *ν_i_^C^* to give the covariance of channel noise^2^. Similarly, Q*_j_^U^* represent covariance components in source space that are scaled by *ν_i_^P^ν_j_^U^* to give the contribution of the *j*-th component to the *i*-th subject's source activity. This scaling by a product of two scale parameters embodies the formal hyperprior that source activity factorises into source and subject-specific components.

Note that the unknown hyperparameters $\lambda_{i}^{•}=\ln\left(\nu_{i}^{•}\right)$ have their own prior densities, $p\left(\lambda_{i}^{•}\right)$, which place non-negative lognormal hyperpriors on the corresponding scale parameters $\nu_{i}^{•}$. In what follows, we assume that *V* is a fixed Gaussian autocorrelation matrix with a smoothness that corresponds to any filtering applied during pre-processing. We will also assume that *Q^C^ *= *I* and select the source components *Q*_1_^*U*^,…,*Q_M_^U^* according to the model *m* required (*e.g*., multiple sparse priors, minimum norm *etc*.). We use weakly informative hyperpriors with *η *= − 16 and *R *= 32*I* that tend to eliminate unnecessary components.

#### Gaussian process models

This model may look complicated but it can be reduced to a very simple form, which is easy to invert. This form is a Gaussian process or covariance function model, which effectively expresses the expected sample covariance of the data as a covariance function of the hyperparameters. Note that in this form, the parameters are eliminated and we have only to optimise the hyperparameters. For a single subject, the sample covariance of spatiotemporally ‘normalised’ responses *S(Y˜_i_*) and its expectation Σ˜(*λ_i_*) are

(3) $$\begin{array}{c} S\left({\tilde{Y}}_{i}\right)=\frac{1}{n}{\tilde{Y}}_{i}V^{-1}{\tilde{Y}}_{i}^{T}\\ \tilde{\Sigma }\left(\lambda_{i}\right)=\nu_{i}^{P}\left(\nu_{1}^{U}{\tilde{Q}}_{1}^{U}+\ldots +\nu_{M}^{U}{\tilde{Q}}_{M}^{U}\right)+\nu_{i}^{C}{\tilde{Q}}_{i}^{C}\\ {\tilde{Q}}_{k}^{U}={\mathrm{GQ}}_{k}^{U}G^{T}\\ {\tilde{Q}}_{i}^{C}=A_{i}Q^{C}A_{i}^{T} \end{array}$$

Essentially, this says that that the predicted covariance of normalised channel data, ${\tilde{Y}}_{i}=A_{i}Y_{i}$ over time, is a mixture of covariance components from source space and measurement space. The normalization matrix *A*_*i*_ = *GL*_*i*_^*T*^(*L*_*i*_*L*_*i*_^*T*^)^− 1^ ⇐ *A*_*i*_*L*_*i*_ = *G* effectively re-samples the channel data to return what would have been observed if the gain-matrix was an arbitrary matrix, *G*. We usually use the gain-matrix from the first subject; *i.e*., *G* = *L*_1_. This re-sampling is useful because it allows us to specify a covariance function model for the sample covariance over subjects

(4) $$\begin{array}{c} S(\tilde{Y})=\sum_{i}S({\tilde{Y}}_{i})\\ \tilde{\Sigma }\left(\lambda \right)={\overset{―}{\nu }}_{1}^{U}{\tilde{Q}}_{1}^{U}+\ldots +{\overset{―}{\nu }}_{M}^{U}{\tilde{Q}}_{M}^{U}+\nu_{1}^{C}{\tilde{Q}}_{1}^{U}+\ldots +\nu_{N}^{C}{\tilde{Q}}_{N}^{U}\\ {\overset{―}{\nu }}_{k}^{U}={\overset{―}{\nu }}^{P}\nu_{k}^{U}\\ {\overset{―}{\nu }}^{P}=\nu_{1}^{P}+\ldots +\nu_{N}^{P} \end{array}$$

Again, the covariance function Σ˜(*λ*) is a mixture of components from source and measurement space; these are a mixture of *M* source-specific spatial components and *N* subject-specific sensor-noise components. In this model, the hyperparameters $\lambda_{k}^{U}=\ln{\overset{―}{\nu }}_{k}^{U}$ encoding contributions from source space are the same over subjects; it is these we seek to provide an empirical prior on source space that is conserved over subjects. We now have a Gaussian process model for a group of subjects that is easy to invert or optimise.

#### Optimising Gaussian process models

Covariance function formulations of linear models are important because their hyperparameters can be optimised using standard covariance component estimation techniques to maximise the model evidence, *p*(*Y*|*m*) for any data *Y* under model *m*^3^. Once the hyperparameters have been optimised the conditional density of the parameters is easy to compute, either for the group average or each subject (see below). In this context, optimisation uses an augmented restricted maximum likelihood scheme (Harville, 1977), as described in Friston et al. (2007). This scheme is formally equivalent to a variational inversion under the mean-field assumption that the conditional density *q*(*J*,*λ*) = *q*(*J*)*q*(*λ*) factorises into Gaussian marginals. Here, *q*(*J*) = *N*(*μ*^*J*^,Σ^*J*^), where *μ*^*J*^ and Σ^*J*^ are the conditional expectation and covariance of parameters; similarly for the hyperparameters, *q*(*λ*) = *N*(*μ*^*λ*^,Σ^*λ*^). Under this Gaussian or Laplace assumption, the hyperparameters of any Gaussian process model maximise a variational free-energy bound on the log-evidence^4^

(5) $$F=-\frac{D}{2}tr\left(\Sigma^{-1}S\right)-\frac{D}{2}\ln\left|\Sigma \right|+\frac{1}{2}\ln\left|\Sigma^{\lambda }\Pi \right|-\frac{1}{2}{\left(\mu^{\lambda }-\eta \right)}^{T}\Pi \left(\mu^{\lambda }-\eta \right)$$

Where, *D* represents the total number of samples used to evaluate the sample covariance matrix^5^ and π = *R*^-1^ is the prior precision of the hyperparameters from Eq. (2). This bound is a function of sample and predicted covariances, *S*(*Y*) and Σ(*μ*^*λ*^) respectively. Optimisation is relatively simple and involves iterating

(6) $$\begin{array}{c} F_{\lambda i}=-\frac{D}{2}tr\left(P_{i}\left(S-\Sigma \right)\right)-\Pi_{ii}\left(\mu_{i}^{\lambda }-\eta_{i}\right)\\ F_{\lambda \lambda ij}=-\frac{D}{2}tr\left(P_{i}\Sigma P_{j}\Sigma \right)-\Pi_{ij}\\ \Delta \mu^{\lambda }=-F_{\lambda \lambda }^{-1}F_{\lambda }\\ \Sigma^{\lambda }=-F_{\lambda \lambda }^{-1} \end{array}$$

until convergence. The matrix *P*_*i*_ is the derivative of the precision, Σ(*μ*^*λ*^)^− 1^ with respect to the *i*-th hyperparameter. *F*_*λ*_ and *F*_*λλ*_ are the gradient and expected curvature of the free-energy. Note that this scheme deals with relatively small *c* × *c* matrices whose size corresponds to the number of channels. In other words, computational load does not scale with the number of time-bins, trials or subjects.

Put simply, this procedure optimises the amount of each covariance component iteratively, to minimise the difference between the sample covariance and the ensuing mixture of components. The difference is essentially the Kullback-Leibler divergence between the sample and predicted Gaussian densities encoded by the covariances (Harville, 1977).

### A two-stage optimisation

The hyperparameters of our hierarchical model can be optimised in two stages. First, we estimate the hyperparameters of Σ˜(λ), using the sample covariance over subjects S (*Y*˜) to furnish the conditional estimates of source-specific scale parameters, ${\overset{―}{\nu }}_{1}^{U},\ldots {\overset{―}{\nu }}_{M}^{U}$. These define an empirical prior covariance on the sources $\overset{―}{U}={\overset{―}{\nu }}_{1}^{U}Q_{1}^{U}+\ldots +{\overset{―}{\nu }}_{M}^{U}Q_{M}^{U}$ that is common to all subjects (to within a scaling factor). Second, we optimise subject-specific hyperparameters using the sample covariance of single-subject data *S*(*Y*_*i*_) and the covariance function model

(7) $$\Sigma_{i}\left(\lambda \right)=\nu_{i}^{P}L_{i}\overset{―}{U}L_{i}^{T}+\nu_{i}^{C}Q^{C}$$

Here, the only unknowns are the subject-specific error covariance and source-prior hyperparameters. These are optimised in exactly the same way as above to give the conditional estimates of *λ_i_ *= {*ν_i_^P^*,ν*_i_^C^*}. These specify the *maximum a-posterior* (*MAP*) estimates of source activity using the matrix inversion lemma.

(8) $$\begin{array}{c} \mu_{i}^{J}=M_{i}Y_{i}\\ \Sigma_{i}^{J}=U_{i}-M_{i}L_{i}U_{i}\text{.} \end{array}$$

*M_i_ *= *U*_*i*_*L_i_^T^*Σ_*i*_^-1^ is a *MAP* projector, where the covariance function Σ_*i*_(*λ*) and empirical prior covariance $U_{i}\left(\lambda_{i}\right)=\nu_{i}^{P}\overset{―}{U}$ are evaluated at the conditional modes of *λ*_*i*_.

Time–frequency contrasts of these subject and trial-specific source reconstructions can then be used to summarise the subject-specific responses to each trial type. These contrasts generally test for specific time–frequency components by defining a temporal subspace of interest (*e.g*., gamma oscillations between 300 and 400 ms after stimulus onset). The contrast matrix can be a simple vector; for example a Gaussian window $W\in R^{n\times 1}$ over a short period of peristimulus time or cover specified frequency ranges (with one frequency per column) over extended periods of peristimulus time (Kiebel and Friston, 2004). The conditional expectation of the energy in a contrast is

(9) $$E\left(J_{i}WW^{T}J_{i}^{T}\right)=M_{i}Y_{i}WW^{T}Y_{i}^{T}{\tilde{M}}_{i}^{T}+\Sigma_{i}^{J}tr\left(W^{T}VW\right)$$

See Friston et al. (2006) for details. The conditional estimates of contrast energy can then be used as summaries of condition-specific responses for each subject and entered into statistical models of between-subject responses in the usual way. Eq. (9) provides more accurate estimates than classical beam-forming approaches because uncertainty about the activity enters the estimate of power or energy in a contrast. In other words, the expected energy is not just the energy of expected activity (as assumed in beam-forming) but involves an extra term that accounts for variability in the expected activity (the second term above). In the next section, we will use the equations above to estimate summary-statistics for evoked responses using a series of different models that are defined by the prior covariance components employed.

### Prior covariance components

For the minimum norm model there is only one prior covariance component that corresponds to the identity matrix, Q_1_*^U^ *= *I*. In this simplest case, the between-subject constraint should make no difference to the within-subject estimates of activity. However, for the ARD and greedy search schemes, which use multiple prior components, the effects of between-subject constraints could be quite profound. The multiple sparse priors we use in this paper are described in Friston et al. (2008a) Briefly, they comprise 256 small patches with compact spatial support in each hemisphere and a further 256 components corresponding to a bilateral deployment of homologous patches. Each patch is formed by diffusing a point source on the cortical mesh, using a graph Laplacian with a diffusion coefficient of 0.6. This coefficient is basically a smoothness parameter and can vary between zero and one. In practice, we do not use the data from all peristimulus times but project the responses onto a temporal subspace using a modified Kaiser criterion. This subspace is defined by singular value decomposition of the temporal responses over trials and subjects. This usually identified about four to eight temporal modes that span more than 95% of the data variance. This temporal subspace also precludes frequencies lower than 1 Hz and higher than 64 Hz. This concludes the theoretical background. In the next section, we evaluate the between-subject constraint under the three different hyperprior models detailed above.

## Comparative analyses

In this section, we present comparative analyses of the source reconstructions at the within and between-subject level. Data were acquired as a part of previously published study (Litvak et al., 2007). The original study examined the effect of combined somatosensory and transcranial magnetic stimulation (TMS) on median nerve somatosensory evoked potentials (MN-SSEP). The MN-SSEP were recorded before and after TMS. Each of eleven healthy volunteers (eight men and three women), aged 20.9 to 44.5 (mean 26.3 ± 7.2 years) participated in five experiments, which differed in the TMS parameters. For the present analysis, we use only MN-SSEP recorded before TMS. Thus, our dataset consisted of five MN-SSEP recordings per subject.

### Data acquisition and pre-processing

The recordings were performed under identical conditions with intervals of several days to one year between sessions. Median nerve stimulation was performed using an electrical stimulator (Digitimer D7AH, Digitimer, Welwyn Garden, Hertfordshire, UK) with a standard stimulation block (cathode proximal), pulse width 200 μs at a frequency of 3 Hz and a stimulation intensity of 300% of the individual perceptual threshold. Eighteen hundred stimuli were delivered. Electroencephalographic (EEG) signals were recorded from the scalp with a 64-channel Quickamp system (Brain Products GmbH, Munich, Germany). The electrodes were placed at extended 10–20 system locations and fixed on the subject's head with an elastic cap (EASYCAP GmbH, Herrsching-Breitbrunn, Germany). The electrode impedance was maintained below 5 kΩ. Electrode positions and anatomic reference points were measured for each experiment with a 3D navigation system (Brainsight, Rogue research, Montreal, Canada). The brain signals were average referenced in hardware, filtered between 0 and 560 Hz and sampled at 2000 Hz.

The data were processed with the SPM software package (http://www.fil.ion.ucl.ac.uk/spm). The continuous EEG data were high-pass filtered (above a cut-off frequency 20 Hz) and epoched between − 50 and 50 ms relative to the median nerve stimulus. Trials were rejected if they contained deflections exceeding 50 μV. Channels were marked as bad if they contained such deflections in more than 20% of the trials. The number of trials retained for analysis was 1545 ± 242. No channels were excluded in 34 out of 55 experiments, 1 channel in 12 cases, 2 channels in 5 cases, 3 channels in 2 cases and 6 channels in 2 cases. Retained trials were averaged to obtain MN-SSEP waveforms and used, in combination with measured positions of the retained sensors and the fiducials, as input for SPM source reconstruction.

We chose this dataset for the present study for two reasons. The first is that MN-SSEP sources have been studied extensively with invasive and non-invasive recordings in humans and animals; they are known to reside in the hand area of the primary somatosensory cortex (S1), principally in Brodmann area 3b, which is located at the posterior wall of the central sulcus; with a weaker and slightly delayed source in adjacent Brodmann area 1, which is located on the top of the postcentral gyrus (Allison et al., 1991). The second reason is that this particular dataset is especially suitable for testing source reconstruction with group constraints. By selecting one of the five experiments from each of the eleven subjects, 5^11^ different group MN-SSEP datasets can be formed. Ideally, source reconstructions and statistical tests performed on these group-data should yield consistent results. By looking at how variable the results are, under different hyperpriors, the utility of these models can be assessed. In what follows, an ‘experiment’ means a single recoding session subtending a subject-specific MN-SSEP and ‘group’ refers to collection of experiments from eleven different subjects.

### Source reconstruction

The somatosensory evoked activity was reconstructed from 10 to 40 ms after stimulation. This reconstruction proceeded under the three hyperprior models: minimum norm (MN), multiple sparse priors optimised with ARD and multiple sparse prior partitions optimised with a greedy search (GS). Note that the ARD and GS implicitly optimise both the empirical priors and the model itself. This is because when a prior component is switched on or off, the form of the model changes. Conversely, minimum norm reconstructions with and without group constraints are identical. Thus ARD and GS reconstruction schemes were repeated with and without the formal constraint that imposes the same empirical spatial prior over subjects. In total, we examined five source reconstruction schemes: MN, ARD, GS, ARD with group constraints (gARD) and GS with group constraints (gGS).

**Fig. 1:**
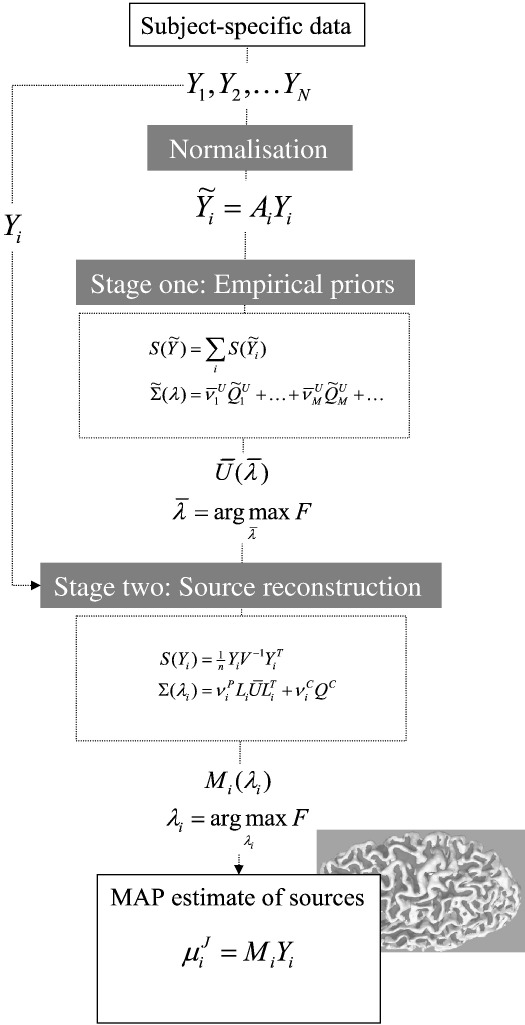
Schematic of the two-stage source reconstruction scheme for group studies. See main text for a detailed explanation of the variables.

Reconstruction without group constraints was performed once for each experiment and each hyperprior model. For reconstruction with group constraints, 100 groups of experiments (each with *N *= 11 subjects) were randomly sampled from the 5^11^ possible groups. Thus, each experiment was used in approximately one fifth of group reconstructions (*i.e*., ∼ 20 reconstructions per hyperprior model). The same 100 groups were used for all reconstruction schemes and *t*-tests (see below). The reconstructions with and without group constraints used exactly the same two-stage procedure but the unconstrained reconstruction used only one subject per group (our software implementation uses just one routine that can be called with one or more subjects). Critically, this means that the final reconstruction (stage 2; in Fig. 1) used just two covariance components (an empirical source prior and sensor noise). This means that models, with and without constraints had the same number of unknown parameters and hyperparameters.

#### Model comparison at the within-subject level

The reconstructed activities per experiment were summarized with a Gaussian time-window centred at 20 ms (with a standard deviation of 8 ms), which is about the time the maximum sensory evoked response is expressed in somatosensory cortex. For each reconstruction, we recorded the free-energy bound on log-evidence (*F*). In the case of reconstructions with group constraints, the values of *F* for each experiment were averaged over all reconstructions in which that experiment was used. The log-evidence provides a measure of the model quality that accommodates both accuracy and complexity (*i.e*., number of priors). Critically, it pertains to the ability of models to explain the data from each experiment but does not reflect those models' generalisability over different datasets. We expected that ARD and GS schemes would provide much better explanations of data, relative to the rather unlikely priors entailed by the MN model. Furthermore, we anticipated that the group constants would *reduce* the log-evidence for ARD and GS, because they constrain the optimisation of subject-specific parameters, when trying to explain a particular experiment. In other words, the advantage of group constraints should only be evident at the between-subject or group level and may even compromise the optimisation at the within-subject or experiment level.

We were interested in relative, rather than absolute values of the free-energy and primarily in the comparison between the GS and ARD schemes, with and without group constraints. Therefore, we analysed the differences between the *F*-values for minimum norm and for the other four schemes for each experiment. We also subjected the *F*-values obtained with GS and ARD to an ANOVA with two factors (hyperprior model; ARD *vs.* GS and between-subject constraint; with *vs*. without). This allowed us to assess the overall effect of different models, group constraints and how the model-effect depends on group constraints.

#### Model comparison at the between-subject level

To assess the quality of reconstructed source activity we performed paired samples *t*-tests using conventional statistical parametric mapping (SPM) procedures to compare the original contrast images and the same images flipped across the midsagittal plane. This is a device to assess the lateralisation of responses evoked by unilateral median nerve stimulation. The contrast images were constructed after diffusion on the canonical mesh and projection into three-dimensional anatomical space.

For each reconstruction scheme 100 *t*-tests were performed using the 100 group-datasets. The statistical images produced by SPM were assessed with respect to two criteria: the maximal *t*-value in each SPM and the distance of this maximum from the maximum in the average SPM, over the 100 replications of the same reconstruction scheme (we will refer to this as the mean location). These two measures, the height of the maximum *t*-value and its distance from the mean location, quantify the sensitivity of the between-subject analysis and its spatial precision. We anticipated that the best model would co-localise reconstructed activity in a consistent way over subjects and produce the largest *t*-values with the least dispersion across repetitions in unilateral somatosensory cortex. To exploit the fact that we had 100 realisations of group data, we present the distribution of these two metrics in terms of their cumulative sample density.

## Results

### Model comparison at the within-subject level

**Fig. 2:**
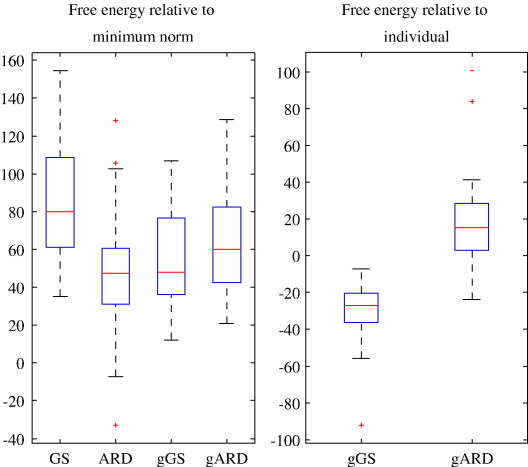
(A) Distributions of the values of free-energy bound to the log-evidence for the four reconstruction methods compared in the present study relative to minimum norm. The distributions are presented as box-plots with lines at the lower quartile, median, and upper quartile values. Values beyond the 1.5 interquartile range from the median are shown as outliers. For gGS and gARD the value for each experiment is an average of ∼ 20 values obtained when that experiment participated in reconstructions with group constraints. (B) Distributions of differences between the average log-evidences and the value obtained for source reconstruction without group constraints.

As expected, the log-evidence for the reconstruction schemes using multiple sparse priors, significantly exceeded the evidence for conventional minimum norm models (mean difference ± SEM GS: 85.4 ± 7.8, ARD: 47.0 ± 7.4, gGS: 56.0 ± 6.4, gARD: 64.2 ± 6.8; Fig. 2A). An analysis of variance of the log-evidences from the experiment-specific inversions showed a significant cross-over interaction between the model and the effect of group constraints (*F*_(1, 54)_ = 258.78, *p* < 0.001). For GS *F* was significantly higher without group constraints (*p* < 0.001) whereas for ARD, *F* was higher with group constraints (*p* < 0.001) (Fig. 2B). On average, the model with the largest evidence was returned by the greedy search without group constraints. For the GS models, this is exactly what we had expected, in that the group constraints compromise the optimisation of the parameters and hyperparameters, in relation to any one dataset. Furthermore, in the absence of group constraints, the superiority of the greedy search over the ARD was not surprising, in that the ARD model is very over parameterised and can easily get stuck in local maxima (see Wipf and Nagarajan, in press for an analysis of convexity of these sorts of objective functions).

What was surprising is the fact that group constraints *improved the evidence for ARD models*. Strictly speaking, this is impossible because any empirical source priors, furnished by group constraints, should have been discovered during the ARD. The only explanation is that ARD finds local maxima in its very high-dimensional search space and that these local maxima are suppressed by group constraints. In other words, the group constraints remove local maxima and allow the ARD scheme to find better solutions. This is encouraging because it means the group constraints must be partly veridical; if they are not, there would have been no improvement in the evidence for ARD models.

Finally, the interaction between model and group constraints makes sense from this perspective; in that ARD benefits much more from constraints than the GS, because it has a much higher dimensional search space to navigate. We were slightly surprised that ARD outperformed GS with group constraints, given our anecdotal experience with these schemes. However, it may be the case that the focal responses evoked by median nerve stimulation do not require the models of distributed responses afforded by the partitions (sets of ARD priors) optimised during the greedy search.

### Model comparison at the between-subject level

**Fig. 3:**
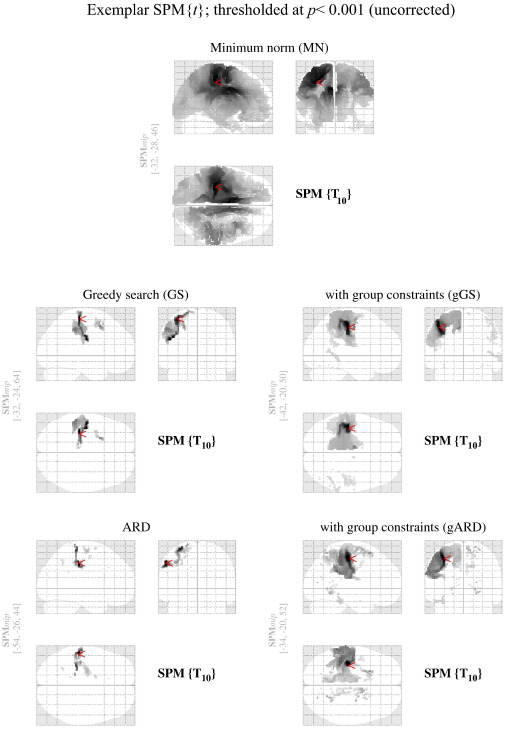
Results of paired *t*-tests comparing the reconstructed images with the midsagittal-flipped version of the same images. All models show maximal activation around the left sensorimotor area. However they differ greatly with respect to the number of suprathreshold voxels. These SPMs have been thresholded at the same level (*p* < 0.001; uncorrected) and the location of the maximum *t*-value is marked by a red pointer in each SPM.

**Fig. 4:**
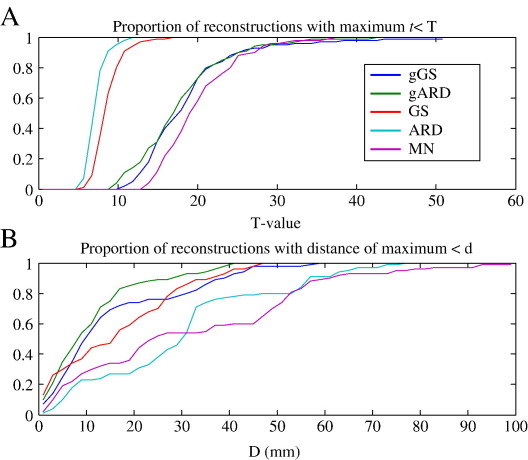
Cumulative distributions of the ‘quality measures’ for the *t*-test SPMs, such as shown in Fig. 2, across 100 repetitions using randomly selected groups of 11 experiments with the same 11 subjects. (A) The maximal *t*-value in the image. (B) The distance of the voxel with the maximal *t*-value from the ‘mean location’ over the 100 group analyses, with the same reconstruction scheme.

Five one-sample *t*-test SPMs testing for a sensory evoked response from one of the randomly selected groups are shown in Fig. 3. These illustrate the similarities and differences across the five reconstruction schemes we evaluated. Fig. 4 shows the cumulative distributions of maximal *t*-values (4A) and the distribution of distance of the voxel with the maximal t-value from the mean location for the same reconstruction scheme (4B).

Interestingly, on average, the maximum *t*-values for the ARD and GS schemes were lower than for minimum norm, but for GS with group constraints the difference was not significant (GS: *p* < 0.001, ARD: *p* < 0.001, GS group: *p* = 0.07, ARD group: *p* = 0.001). Repeated measures two-way ANOVA^6^ showed no interaction between the hyperprior model and the effect of group constraints (*F*_(1, 99)_ = 0.52, *p* = 0.47). Main effects of both factors were significant (hyperprior type: *F*_(1, 99)_ = 7.78, *p* = 0.006, group constraints: *F*_(1, 99)_ = 512.5, *p* < 0.001). Maximal *t*-values were higher for GS, than for ARD and higher with group constraints, than without. The highest *t*-values (except for minimum norm) were attained with GS with group constraints. However, the difference between GS with group constraints and ARD with group constraints was not significant (*p* = 0.29).

It might seem paradoxical for the minimum norm solutions to yield the highest *t*-values, given their models had the lowest evidence on a per subject basis. The reason for this is that the MN solutions deploy reconstructed activity in a non-focal fashion, with very little spatial specificity. This is evident when we consider the evaluation of spatial precision: The dispersion of maxima under GS (*p* < 0.001), gGS (*p* < 0.001) and gARD (*p* < 0.001) was much smaller than for minimum norm models. ARD without group constraints was not significantly different from minimum norm, with respect to this measure (*p* = 0.55). The cumulative densities in Fig. 4B shows that 50% of the SPMs obtained with GS under group constraints furnished a maximum within about 10 mm of the mean location. This contrasts with the MN solutions, where 50% of maxima were only within about 30 mm of their mean location. ANOVA showed a significant interaction between the model and the effect of group constraints (*F*_(1, 99)_ = 42.01, *p* < 0.001). For GS there was no significant effect of the group constraints on dispersion (*p* = 0.6). For ARD, however, the effect of group constraints was highly significant (*p* < 0.001). The best results (lowest distance) were attained using ARD with group constraints. These results were significantly better than for GS with group constraints (*p* = 0.008). Again, ARD benefits significantly more from group constraints, relative to GS. In fact, in terms of spatial precision it outperformed GS (but not in terms of sensitivity), provided it has access to group constraints. As above this may reflect the focal nature of sensory evoked potentials in this paradigm.

## Discussion

Source reconstructions with multiple sparse priors (Friston et al., 2008a) yield solutions which are much more focal than those provided by classical minimum norm models. Furthermore, they have significantly higher model evidences in relation to single-subject data. However, the regional specificity of these solutions turns into a disadvantage when contrast images are pooled across different experiments. This is because inter-subject variability in regional effects can lead to low *t*-values, when sources do not overlap. One way to solve this problem is through spatial smoothing of reconstructed images. However this compromises spatial resolution and subverts the main advantage of multiple sparse priors. Our present study shows that introduction of group constraints makes it possible to attain *t*-values close to those of minimum norm (Fig. 4A); with solutions that remain focal and consistent across subjects (Fig. 4B).

Group constraints may lead to a reduction of model evidence for individual subject data. In the present study, however, this was observed only for GS. For ARD the evidence was actually higher with group constraints (Fig. 2B), suggesting exploration of hyperparameter or model space was somehow finessed by information from other subjects. On comparing the two methods with group constraints, GS and ARD, we conclude that both yield similar *t*-values (close to those of minimum norm case). The largest *t*-values were provided by GS with group constraints; however, for ARD with group constraints the solutions were, anatomically, more consistent across replications. ARD benefited much more than GS from group constraints and this may reflect the large number of hyperparameters it has to optimise. Without group constraints GS is clearly better according to all the three criteria: model evidence, *t*-values and spatial precision.

## Conclusion

We have described a simple device that allows one to place constraints on reconstructed source activity in the context of group studies. This is not a full hierarchical inversion but finesses single-subject source reconstruction; as a prelude to summarizing subject-specific responses for classical inference at the group level. Our analyses suggest that this between-subject constraint markedly increases the reliability of detecting systematic responses over subjects, in terms of their functional anatomy. The inversion procedure is based upon generic variational techniques and yet proceeds quickly and efficiently, using only second-order statistics in channel space. This means the entire inversion for several subjects takes seconds (as opposed to minutes).

## Footnotes

1 We will treat trials and subjects as interchangeable for simplicity. In practice, one would normally invert several trial types (*i.e*., conditions) from several subjects by holding the subject-specific quantities constant over trials.

2 The scheme can be extended easily to model multiple covariance components for channel noise.

3 Here, a model *m* is defined in terms of the number and form of covariance components.

4 This is formally related to the restricted maximum likelihood (ReML) objective function (Harville, 1977) and is essentially the Kullback-Leibler divergence between the observed and expected Gaussian densities (ignoring hyperpriors and constants).

5 For the single subject *D* = *n* and for the group, *D* = *nN*.

6 Strictly speaking the *t*-value summary statistics cannot be treated as independent summaries, over the 100 realisations of the group analyses, because these realisations borrowed data from the same five experiments in each subject. However, we appeal to the sphericity of mildly correlated but uniform correlations, among the ensuing errors over groups, to eschew a correction for non-sphericity. Heuristically, each of the 100 distinct combinations of the five experiments brings a degree of freedom to the inference about effects over groups.
